# Mild encephalitis/encephalopathy with a reversible splenial lesion (MERS) associated with bacteria meningitis caused by listeria monocytogenes: A case report

**DOI:** 10.1097/MD.0000000000011561

**Published:** 2018-07-27

**Authors:** Jialu Xu, Feng Gao, Zhefeng Yuan, Lihua Jiang, Zhezhi Xia, Zhengyan Zhao

**Affiliations:** Children's Hospital, Zhejiang University School of Medicine, Hangzhou, Zhejiang Province, China.

**Keywords:** hyponatremia, IL-6, listeria monocytogenes, mild encephalitis/encephalopathy with a reversible splenial lesion

## Abstract

**Rationale:**

Mild encephalitis/encephalopathy with a reversible splenial lesion is a clinico-radiological syndrome mainly triggered by viral infection. Bacteria, like listeria monocytogenes, are relatively rare pathogens.

**Patient concerns:**

A two and a half years old girl with a 3-day history of fever and vomiting, complicated by a sudden seizure. She was in a coma after seizure.

**Diagnoses:**

Listeria monocytogenes was detected in cerebrospinal fluid cultures. Serum IL-6 remarkably elevated, and hyponatremia appeared on day 2 of hospitalization. Magnetic resonance imaging of the brain performed on day 3 of hospitalization showed right subdural effusion and a lesion in the central portion of the splenium of the corpus callosum.

**Interventions:**

We administered antimicrobial therapy, intravenous mannitol and hypertonic fluid therapy.

**Outcomes:**

Her neurological symptoms improved gradually. The lesion in the splenium of the corpus callosum completely disappeared on magnetic resonance imaging on day 10 of hospitalization.

**Lessons:**

We diagnosed this case as mild encephalitis/encephalopathy with a reversible splenial lesion caused by listeria monocytogenes. The patient recovered completely clinically and on imaging, without any specific immunomodulatory treatment. It also indicated IL-6 may play a role in the forms of hyponatremia in mild encephalitis/encephalopathy with a reversible splenial lesion.

## 1 Introduction

Mild encephalitis/encephalopathy with a reversible splenial lesion (MERS) is defined as a clinicoradiological syndrome with acute encephalopathy preceded by an acute inflammatory disease.^[1]^ Brain magnetic resonance imagings (MRIs) show transient changes mainly in the corpus callosum and mostly recover without special treatment. This syndrome is mostly reported in children especially in Japan and East Asia. Here, we present a 2.5-year-old girl with MERS caused by listeria monocytogenes.

## 2 Case presentation

A previously healthy 2 and a half years old girl was admitted to our hospital with a 3-day history of fever and vomiting, complicated by a sudden seizure of half a minute on the next day of admission. On admission, she had a temperature of 37.9°C, with neck resistance, but was negative of Kernig sign, Brudzinski sign, and Babinski sign. She was in a coma after seizure and had a Glasgow Coma Scale score of 5 (eyes 1, verbal 1, motor 3).

Laboratory blood testing showed leucocyte count 21,090 (4000–12,000) cells/μL, serum sodium 133 (135–145) mmol/L, and C-reactive protein 180 (0–8) mg/L. Serum interleukin (IL)-6 was 291.4 (1.7–16.6) pg/mL and IL-10 4.1 (2.6–4.9) pg/mL. Serum sodium fell to 118 (135–145) mmol/L on day 2 of hospitalization. Cerebrospinal fluid (CSF) examination revealed leukocytes 96 (0–10) cells/μL, with 60% mononuclear cells, protein 1.6 (<0.45) g/L, and glucose 6.27 (2.78–4.50) mmol/L. So, the primary diagnosis of this patient was bacteria meningitis and hyponatremia.

**Figure 1:**
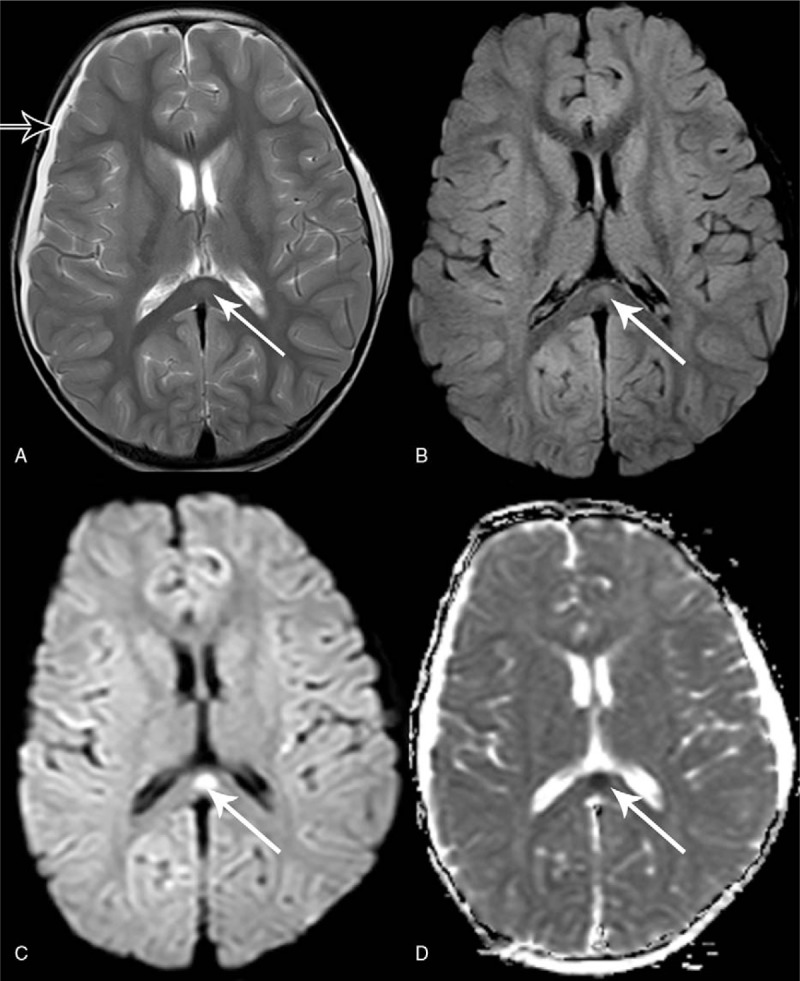
Cranial MRI showing right subdural effusion on T2 (A) and focal hyperintensity signal in the SCC on T2 (A), FLAIR (B), DWI (C), and diffusion restriction on ADC mapping (D). Hollow arrow showed right subdural effusion in (A). White arrows showed the lesion in the SCC on different images in (A, B, C, D).

Cranial MRI was performed on day 3 of admission (6 days after her symptoms began) and showed right subdural effusion on T2-weighted image and a marked hyperintense lesion in the splenium of the corpus callosum (SCC) on T2-weight, fluid-attenuated inversion recovery (FLAIR) images, and diffusion-weighted images (DWIs) with a reduced apparent diffusion coefficient (ADC) mapping (Fig. 1). According to the change of the cranial MRI, we made the supplementary diagnosis of bacteria meningitis with subdural effusion and MERS. On the fourth day of admission, listeria monocytogenes was detected in CSF cultures.

**Figure 2:**
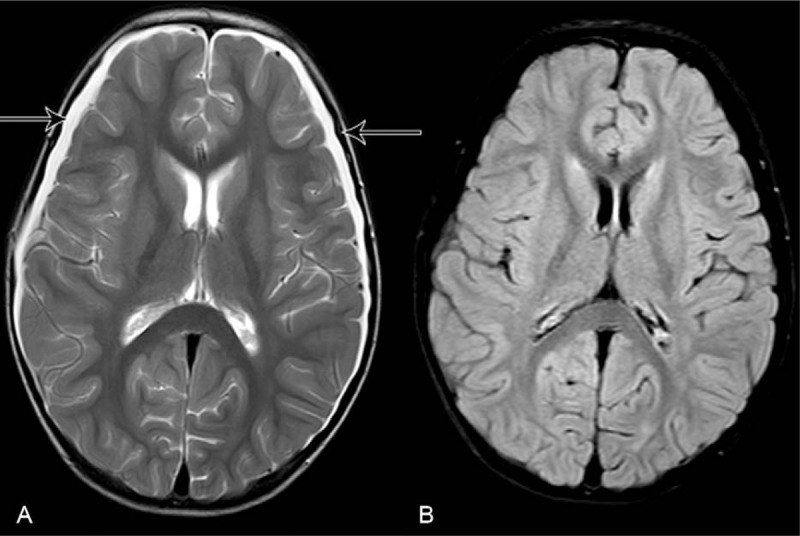
Cranial MRI showing bilateral subdural effusion on T2 (A) and the splenial lesion had completely disappeared on T2 (A) and FLAIR image (B). Hollow arrow showed bilateral subdural effusion in (A).

**Figure 3:**
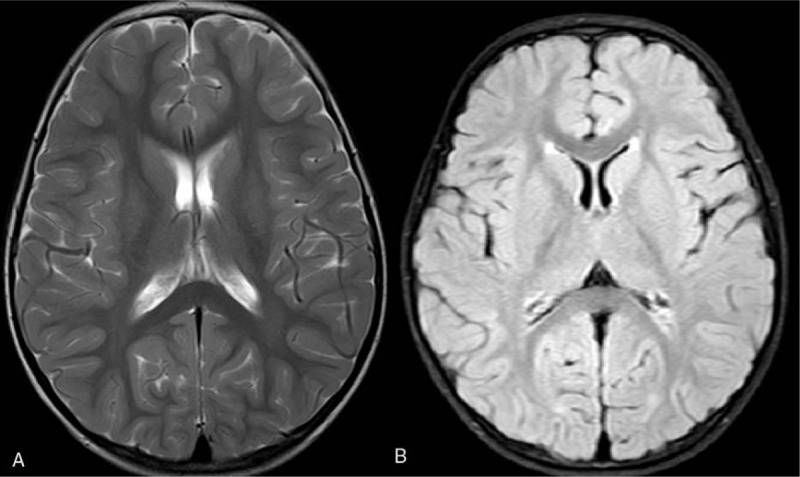
Cranial MRI showing the subdural effusion had completely absorbed on T2 (A) and FLAIR image (B).

The patient received antibiotics treatment first with panipenem/vancomycin, which was then switched to ampicillin and vancomycin after listeria monocytogenes was detected. Also, intravenous mannitol and hypertonic fluid (3% sodium chloride) therapy were started. The patient clinical condition improved over the subsequent 7 days, with gradual resolution of her symptoms. She showed normal mental status after 7 days of hospitalization. Her Glasgow Coma Scale score reached 15 (eyes 4, verbal 5, motor 6). Serum sodium raised to 134 (135–145) mmol/L on day 4 of hospitalization. Re-examination of the cranial MRI on day 10 of hospitalization (13 days after his symptoms began) showed bilateral subdural effusion on T2-weighted image, but the splenial lesion had completely disappeared on T2 and FLAIR image (Fig. 2). The patient performed well on 3 months postdischarge follow-up and the cranial MRI showed the absorption of subdural effusion (Fig. 3).

## 3 Discussion

MERS is characterized by a reversible lesion with homogenously reduced diffusion in the corpus callosum. The most common neurological symptom is delirious behavior, followed by consciousness disturbance, and seizures.^[2]^ All of these clinical symptoms recover within a month.^[3]^

Cranial MRI shows representative features of MERS as prolonged signal on T2, reduced diffusion on DWI, restricted water diffusion, and decreased ADC values in the SCC.^[3]^ The lesion on MRI could completely disappear on follow-up. Moreover, MERS could be classified into a transient lesion in SCC called type I or associated in the frontal white matter called type II.^[4,5]^

We find listeria monocytogenes, a new infectious agent of MERS. The most common pathogens of MERS are viruses, and rarely bacteria, which are reported to constitute 3.3% of all causes of MERS,^[6]^ including *Streptococcus pneumonia*, Staphylococcus^,^*Escherichia coli*, and *Enterococcus faecalis*.

The pathogenesis of MERS remains unclear. Many reports showed that MERS is associated with hyponatremia. Takanashi et al^[7]^ reported that most patients with MERS had mild hyponatremia (131.0 ± 4.1mmol/L). Cerebral edema caused by an electrolyte/water imbalance might be an underlying pathophysiology of MERS.

We observed a transcient hyponatremia that serum sodium fell to 118 mmol/L as well as the remarkable elevation of serum IL-6 at the beginning of the disease on our patient. A recent study found a role for IL-6 as a second messenger acting as an effector in brain areas involved in vasopressin release. This mechanism plays a role in significant forms of hyponatremia.^[8]^

CSF IL-6 levels were significantly higher in cases of bacterial meningitis, which was well beyond the normal values and those found in patients with aseptic meningitis.^[9]^ Several animal experiments showed IL-6 levels in CSF in sheep with encephalitic listeriosis were significantly higher and IL-10 level was significantly lower in nonsurvivors than survivors.^[10]^ The quantities of Listeria-induced IL-6 in infected mice directly correlate with the severity of the infection.^[11]^ Although we did not test the level of IL-6 in CSF, but the remarkable increase of serum level of IL-6 also hints the severity of the infection.

Patients with MERS caused by different pathogens are generally reported to recover completely both clinically and on imaging.^[12–14]^ Only some patients with type II lesions can develop neurologic sequelae.^[4]^ All the clinical symptoms of our patient recovered within 7 days and the splenial lesion had completely disappeared in 10 days. Subdural effusion is one of the commonest intracranial complications of purulent meningitis.^[15]^ Little subdural effusion also occurred coinstantaneous in our patient and it finally absorbed in 3 months.

Although there is no any treatment guideline for MERS, given its generally benign prognosis, specific immunomodulatory treatment may not be justified.^[16]^ That means there is no obvious supporting evidence of treatments with steroids or intravenous immunoglobulin (IVIG) for MERS.^[3,13,14]^

## Acknowledgment

The authors would like to thank Mr Xinghui Yang for excellent technical support.

## Author contributions

**Investigation:** Zhefeng Yuan.

**Supervision:** Zhezhi Xia.

**Validation:** Lihua Jiang.

**Writing – original draft:** Jialu Xu.

**Writing – review & editing:** Feng Gao, Zhengyan Zhao.
